# Prolonged Inflammation and Infectious Changes in the Corneal Epithelium Are Associated with Persistent Epithelial Defect (PED)

**DOI:** 10.3390/pathogens12020261

**Published:** 2023-02-06

**Authors:** Tanmoy Dutta, Jyoti Sangwan, Moumita Mondal, Mehak Vohra, Vatsala Nidhi, Abha Gour, Neha Kapur, Nidhi Gupta, Tuhin Bhowmick, Arun Chandru, Umang Mathur, Virender Singh Sangwan, Manisha Acharya, Anil Tiwari

**Affiliations:** 1 Dr Shroff’s Charity Eye Hospital, Cornea and Stem Cells Department, Delhi 110002, India; 2 Institute of Medicine, Sahlgrenska Academy, Gothenburg University, 41345 Gothenburg, Sweden; 3 Pandorum Technologies Pvt. Ltd., Bangalore 560065, India

**Keywords:** persistent epithelial defect (PED), re-epithelialization, interlukin-6, inflammation, correlation

## Abstract

**Purpose:** Failure of rapid re-epithelialization within 10–14 days after corneal injury, even with standard supportive treatment, is referred to as persistent corneal epithelial (CE) defect (PED). Though an array of genes regulates reepithelization, their mechanisms are poorly understood. We sought to understand the network of genes driving the re-epithelialization in PED. **Method:** After obtaining informed consent, patients underwent an ophthalmic examination. Epithelial scrapes and tears samples of six PED patients and six individuals (control) undergoing photorefractive keratectomy (PRK) were collected. RNA isolation and quantification were performed using either the epithelial scrape taken from PED patients or from HCLE cells treated with control tears or tears of PED patients. Quantitative real-time polymerase chain reaction (qRT-PCR) was performed to detect the expression of a few important genes in CE homeostasis, inflammation, and cell–cell communication, viz., Kruppel-like factor 4 (KLF4), GPX4, IL6, TNFα, STING, IL8, desmoglein, and E-cadherin, among others. Their expressions were normalized with their respective housekeeping genes and fold changes were recorded. KLF4 localization and MMPs activity was carried out via immunofluorescence and zymography, respectively. **Results:** KLF4, a transcription factor important for CE homeostasis, was upregulated in tears-treated HCLE cells and downregulated in PED patients compared to the healthy PRK group. Cell–cell communication genes were also upregulated in tears-treated cells, whereas they were downregulated in the PED tissue group. Genes involved in proinflammation (IL6, 282-fold; TNFα, 43-fold; IL8, 4.2-fold) were highly upregulated in both conditions. MMP9 activity increased upon tears treatment. **Conclusions:** This study suggests that tears create an acute proinflammatory milieu driving the PED disease pathology, whereas the PED patients scrapes are an indicator of the chronic stage of the disease. Interferons, pro-inflammatory genes, and their pathways are involved in PED, which can be a potential target for inducing epithelialization of the cornea.

## 1. Introduction

Vision impairment is a serious threat worldwide, which is more rampant in developing countries such as India. As reported by the World Health Organization, 2.2 billion people globally suffer from vision impairment [1]. Corneal opacity is one of the major reasons for vision impairment and preventable blindness [2]. Due to a lack of awareness and the unavailability of nearby ophthalmic clinics, many people show up at the clinic very late, at which point their condition is difficult to treat. However, even with standard care and treatment, patients often do not heal.

The corneal epithelium (CE) is a self-rejuvenating stratified squamous epithelial tissue at the most anterior part of the eye that serves as a transparent barrier, enabling clear vision [3]. The structure and functions of CE are controlled by complex cues that mediate genetic and epigenetic changes [4,5]. During development, CE stratification involves a perfectly choreographed balance between cellular proliferation and differentiation that continues during adult CE homeostasis, when additional demands due to cell loss from periodic sloughing off the superficial cells and epithelial scrape injuries need to be met [6,7].

The transparent CE maintains corneal clarity by protecting the eye against infection and damage and facilitating nutrient transfer and gaseous exchange from tear fluid. A breach of CE integrity, resulting from mechanical trauma, infection, keratopathy, dry eye, systemic and genetic disorders, and limbal stem cell deficiency (LSCD), causes persistent corneal epithelial defects (PEDs) [8], which result in corneal scarring; ulceration; neovascularization; conjunctivalization, ultimately leading to corneal opacification; and visual loss. Clinically, failure of rapid re-epithelialization within 10–14 days after a corneal injury, even with standard supportive treatment, is referred to as a persistent corneal epithelial defect (PED) [9,10].

There are no definitive epidemiological studies or prevalence data available in India on PED. The annual incidence of PED is less than 200,000 cases in the U.S. The incidence of PED following a corneal transplant is around 7558 cases per year in the USA [7]. Though there are PEDs of different etiology, clinical treatment remains limited and somewhat similar.

In this study, we aimed to investigate the molecular changes in the corneal epithelium and tear samples of PED patients. To achieve this, PED patients with the etiology of infection were specifically recruited. This was carried out: (a) to reduce the chance of sample heterogeneity, (b) to exclude the fact that the severity of chemical or mechanical injury might be dependent on the exposure and amount of the chemicals or level of trauma in the eyes. In case of viral or bacterial infection in PED, the host factors play a major role, yet the mechanism is poorly understood. Tears contain many secreted factors and enzymes, which are altered when there are any external forces, including infections, in the eyes [11,12]. However, there is a lacuna of knowledge on how this diseased tear can influence the corneal epithelial cells. We thus wanted to investigate how the healthy vs. diseased tears from healthy individuals or PED patients affect the corneal epithelial cells in vitro. Additionally, the in vivo gene expression levels in the corneal epithelium of healthy or PED patients were measured using qPCR from the corneal epithelial scrapes. Further, we used gene expression data and clinical information to establish a clinicopathological correlation.

We hypothesized that tears from diseased eyes might contain crucial factors for infection and non-healing factors of epithelial defects, which can create a feedback loop and further deteriorate the process. We were interested to see how tears from PED patients can affect the corneal epithelial cells and how their expression correlates with in vivo gene expression results. We also investigated if the secreted factors in tears and the corneal epithelial cells synergistically affect corneal healing or if they act independently by stimulating different molecular mechanisms.

## 2. Materials and Methods

### 2.1. Study Design

The study protocol was approved by the Institutional Review Board of Dr. Shroff’s Charity Eye Hospital, Delhi. Persistent corneal epithelial defect (PED) patients with active infections (of viral or bacterial etiology) and age-matched healthy individuals undergoing photorefractive keratectomy (PRK), were recruited for this study from this single tertiary care eye hospital. All participants were included in this study with signed informed consent. All study participants underwent thorough ophthalmic examinations, slit lamp examination with fluorescein dye to evaluate the epithelial defect, and anterior segment-optical coherence tomography (AS-OCT) to evaluate corneal thickness and opacity. The etiology of the infection was determined by an expert clinical team of the Cornea department in the tertiary care unit of Dr. Shroff’s Charity Eye Hospital and by microbiological tests. The tear samples were collected from the study participants using a Schirmer’s strip. Tears were collected up to the 25 mm mark or for a maximum of 5 min and collected in a sterile empty microcentrifuge tube and immediately processed or stored at −20 degrees. Epithelial scraping, which is routinely followed in the clinic for PED patients, was collected from all PED patients and stored in RNA preserving solution. Similarly, corneal epithelial tissue was collected from individuals undergoing PRK surgery for correction of refractive errors and stored in RNA.

### 2.2. Cell Culture and Maintenance

Human Cornea limbal epithelial cells (HCLE) (a kind gift from Dr. Ilene Gipson, Harvard Medical School, Boston, MA, USA) were cultured in complete culture media as described previously [13] (Keratinocyte- serum free media (KSFM), supplemented with Bovine pituitary extract and 0.2 ng/mL Epidermal Growth factor (EGF)). HCLE cells were passaged at a 70% confluent stage.

### 2.3. Extraction of Tears Protein and HCLE Treatment

Total proteins were extracted from the tear samples in sterile 200 uL of 1X PBS and filtered using 0.2-micron filters. Crude proteins were quantified using 280 nm absorbance in NanoDrop (Thermo Fisher Scientific, Waltham, MA, USA). To treat HCLE, cells were seeded at a density of 50,000 cells/well of a 12-well plate in complete media for 24 h. Cells were treated with an equal concentration of isolated tears for 48 h. At the end of the experiment, RNA was isolated from HCLE cells using a Qiagen Mini-RNA isolation kit (Cat. No./ID: 74004), according to the manufacturer’s protocol and quantified using NanoDrop. An equal amount of RNA from the PED and PRK individuals was converted to cDNA using the Verso cDNA synthesis kit. Quantitative Real-time polymerase chain reaction (PCR) (qRT-PCR) was performed in duplicate for all the samples using SYBR green dye on an Azure qPCR instrument (Azure Biosystem, Ceilo 6, Dublin, CA, USA). RPL10 and beta-actin were taken as housekeeping genes and their geometric mean was used for data normalization. All results are shown in terms of relative expression (ΔΔCt) of genes, where PRK is the control group and PED is the disease group.

### 2.4. Gelatin Zymography

To evaluate the levels of active MMPs in the supernatant of the HCLE treated with tears extract, gelatin Zymography was performed using 8% acrylamide gel with a final concentration of 1 mg/mL gelatin. An equal concentration of cell soup was mixed with 4x loading eye (no reducing agent and no heating) and incubated for 5 min. An equal amount of protein and pre-stained protein standards (Thermo Fischer Scientific, Waltham, MA, USA, Cat no. 26616) was loaded in each well of the gel and separated via electrophoresis. Next, the gel was incubated in 1% triton-X 100 for 30 min twice, followed by incubation in developing buffer (composition) overnight at 37 °C. The next day, developing buffer was removed and the gel was stained with Coomassie blue stain for 10 min. The gel was washed and incubated in de-staining buffer to remove the excess stain. The clear band in the gel indicates the MMPs activity. The bands were quantified using ImageJ software and were plotted as a bar graph.

### 2.5. Immunofluorescence

For immunofluorescence, HCLE cells were seeded on coverslips in complete media. Cells were treated with tear extracts for 48 h. At the end of the experiment, cells were washed once with PBS, followed by fixing with 4% paraformaldehyde, permeabilization with 0.1% triton-X 100 for 5 min, and washed 3 times with PBS. Blocking was carried out using 5% bovine serum albumin (BSA) for 1 h at room temperature followed by adding KLF4 antibody (CST, 1:100) and incubation overnight at 4 °C. Cells were washed 3 times with PBS and secondary antibody (Invitrogen, Waltham, MA, USA, Goat anti-rabbit, Alex flour 468) was added (1:200) and incubated for 1 h at RT. Cells were washed with PBS 3 times. Coverslips were mounted using VECTASHIELD^®^ Antifade Mounting Medium H-1000-10 and were imaged using a Zeiss inverted fluorescent microscope.

### 2.6. Data Analysis

qPCR data analysis was conducted in Azure Cielo Manager software and Microsoft Excel. Plots were generated using GraphPad Prism (version 9.4.1, California, USA), and R programming (version 4.0, New Zealand). In graphical presentations, normalized fold change with standard errors of mean (SEM) was used for qPCR data. Outliers in the in vitro experiment were removed for data analysis. The band intensity of the Zymogram was measured in ImageJ software (1.53k version, NIH, USA). Possible protein–protein interactions and biological pathways were determined using the String database [14].

### 2.7. Correlation Plot

A correlation plot was made using the R built-in functions, ‘Hmisc’, and ‘corrplot’, packages. Relative mRNA expression values, i.e., ΔΔCt, and quantitative clinical data, i.e., age, duration of PED at the time of sample collection and size of the defect (mm^2^) were used to generate the plot. Pearson’s correlation test was performed.

## 3. Results

### 3.1. Ophthalmic and Clinical Evaluations

**Figure 1:**
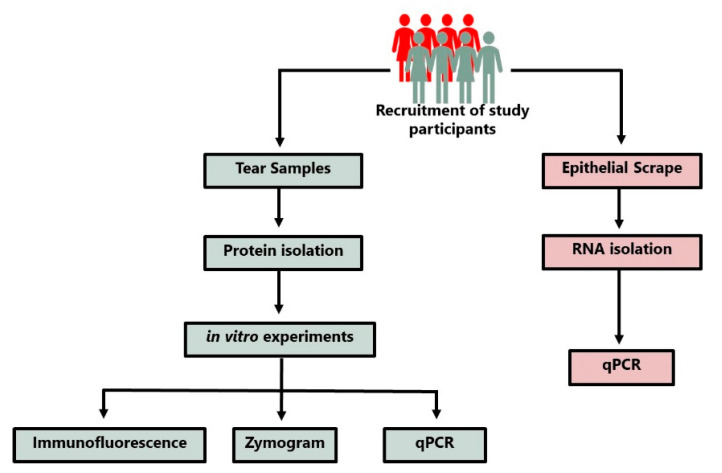
Schematic depicting the methodology of sample collections and the experimental design.

**Figure 2:**
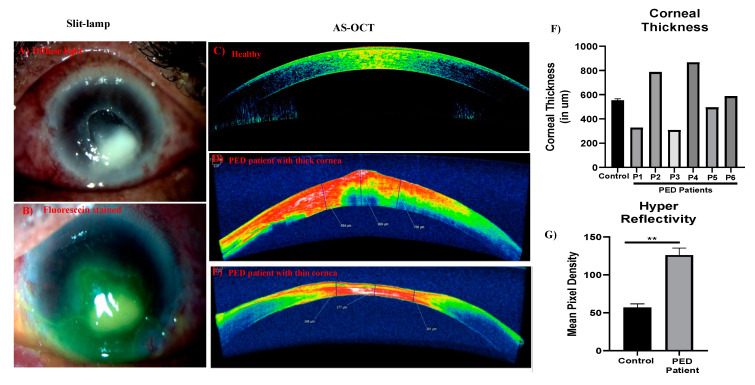
Clinical diagnosis and ophthalmic evaluation. Slit lamp images showing the corneal ulcers associated with PED, diffuse light showing the non-healing ulcers (**A**), and the same region stained with fluorescein (**B**) to show the lack of epithelium. AS-OCT image of a healthy individual (**C**) and representative images of PED patients (**D**,**E**). Corresponding quantitative bar graph showing the corneal thickness (**F**) and hyperreflectivity, measure of opacity (**G**). ** indicates statistical significance *p* ≤ 0.05.

The study was approved by the IRB committee of Dr. Shroff’s Charity Eye Hospital (SCEH), Delhi, India. All patients were recruited after obtaining informed consent. Patients presented at SCEH were examined by expert clinicians and were diagnosed with PED. Age matched controls were the individuals undergoing PRK at Dr. Shroff’s Charity Eye Hospital, Delhi. Figure 1 shows the overall methodology and experimental design of this study. All PED patients had infections identified clinically by expert clinicians; patients had herpes simplex virus (HSV) keratitis and *Pseudomonas* infections (patient summary in Table 1). Figure 2 shows the representative images of PED ophthalmic evaluation, diffuse lamp, and fluorescein dye, indicating the non-healed ulcers in PED. AS-OCT indicates the thickness of the cornea and the extent of opacity as measured by hyperreflectivity score. Ophthalmic evaluation revealed that PED patients have variable corneal thickness with increased hyperreflectivity (opacity) compared to the control (Figure 2).

### 3.2. PED Tears Create an Acute Inflammatory Milieu That Possibly Compromises the CE Homeostasis

**Figure 3:**
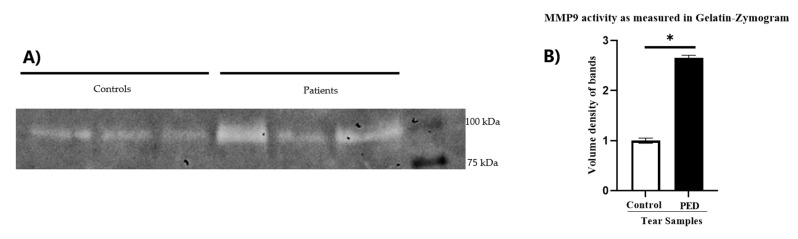
Evaluation of the levels of active MMPs in the culture soup of HCLE cells treated with PED patient tears and control tears. Gelatin zymogram showing the levels of MMP9 in controls and PED patient tears (**A**). The corresponding histogram showing the quantitative volumetric density of the bands, as detected in gelatin zymography (**B**). * Statistically significant according to *t*-test, *p* < 0.05, n = 3.

To test the effect of tear samples in active PED patients in vitro, Human Corneal Limbal Epithelial (HCLE) cells were cultured with extracted proteins from the tear samples of three PED patients as well as healthy individuals. We hypothesized that this may be due to the hyper secretion of matrix metalloproteases (MMPs), which degrades different extracellular matrix (ECM) proteins in case of infections [15]. To test the hypothesis, we checked the level of MMPs, that are most abundant in infections, by performing a gelatin zymogram in the HCLE cells treated with tears extracts condition media (Figure 2). Results showed that PED-induced HCLE cells had significantly higher MMP9 activity compared to cells treated with healthy tears. (Figure 3), indicating that PED tear samples have factors that can induce the expression of activated MMPs. High MMPs possibly hinder the epithelialization phase [16] by degrading the cell–cell adhesion, which is an important part of the healing process, and the extracellular matrix responsible for cell–cell communications and basement membrane.

**Figure 4:**
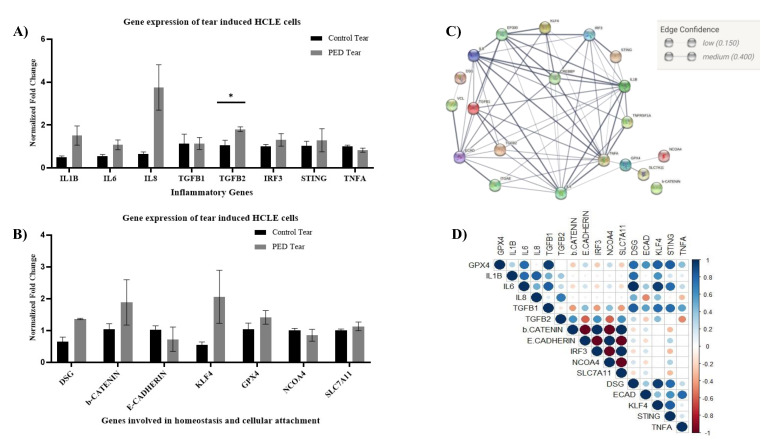
Gene expression in the HCLE cells treated with the control and PED patients, the STRIN network analysis, and the correlation plot. Error bars represent standard error of means. A *t*-test was performed (* implies significantly different, *p* < 0.05), IL1B—Interleukin 1-beta; IL6—Interleukin 6; Il8—Interleukin 8; TGFB1—Transforming growth factor-beta (TGFβ) 1; TGFB2—Transforming growth factor-beta 2; IRF3—Interferon regulatory factor 3; STING—Stimulator of interferon genes; TNFA—Tumor necrosis factor-alpha. mRNA level changes in the HCLE cells as detected by qRT-PCR (**A**). Error bars represent standard error of mean. DSG—desmoglein, b-Catenin—beta Catenin; KLF4—Kruppel-like factor 4, GPX4—Glutathione peroxidase 4, NCOA4—Nuclear Receptor Coactivator 4; SLC7A11—Solute Carrier Family 7 Member 11 (**B**). The STRING analysis of the same gene dataset used to identify the interactions with other genes (**C**). Correlation plot showing positive and negative correlation with each other (**D**).

As increased MMPs activity is associated with a pro-inflammatory state of the tissue, we evaluated the gene expression of pro-inflammatory cytokines in HCLE cells treated with tear extracts. Inflammation is one of the crucial phases in the wound-healing process. Though it is required to combat infections, prolonged inflammation leads to persistent and non-healing wounds. Thus, we looked for a few important inflammatory cytokines known to be involved in the cornea. We looked for mRNA level expression of TNF-α, IL1-beta, IL6, IL8, TGFβ1, TGFβ2, STING, and IRF3 using quantitative polymerase chain reaction (qPCR) (Figure 4A). Most of the genes, viz., IL1-beta, IL6, IL8, IRF3, and TGFβ2 showed elevated expression in PED compared to the healthy group. TGFβ2 had significantly higher expression in the PED group (n = 3, *p* < 0.05, *t*-test).

**Figure 5:**
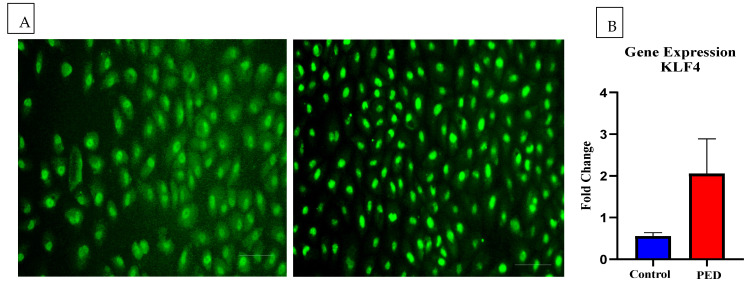
Immunofluorescent images showing the expression and localization of KLF4 in the control and PED tears-treated HCLE cells. HCLE cells treated with control and PED tears followed by staining with anti-KLF4 antibody. Scale bar: 100 µm (**A**). Bar graph shows the expression of KLF4 at the transcript level by qRT-PCR (**B**).

**Figure 6:**
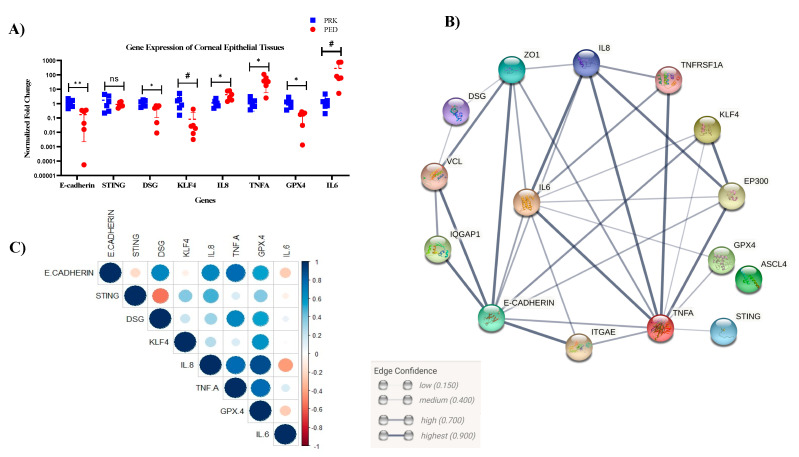
Gene expression relation and corresponding STRING analysis and correlation plot of the PED epithelial scrape. qRT-PCR showing the gene expression profile in the PED and control group (**A**). STRING analysis for the same set of genes, showing the potential interacting partners and their edge coefficient (**B**). Correlation plot and the confidence levels of the same gene set (**C**) n = 6; test—*t*-test (* *p* < 0.05; ** *p* < 0.01, # *p* < 0.1; ns non-significant).

Similarly, a few important genes involved in cell–cell communication and corneal epithelial homeostasis, viz., desmoglein, E-cadherin, beta-catenin, GPX4, NCOA4, SLC7A11, and KLF4 were also looked for in the tear-induced HCLE cells (Figure 4B). Interestingly, the expression of KLF4 was higher in the PED-tear group, though statistically not significant. KLF4 is an important transcription factor and plays a crucial role in maintaining CE homeostasis by regulating epithelial–mesenchymal transitions, plane of cell division, cell cycle, etc. However, to further confirm the gene expression-level findings, immunofluorescence staining was performed with an anti-KLF4 antibody. It was observed that in PED-tear-induced cells, KLF4 was localized in the nucleus, whereas healthy-tear-induced cells showed the presence of KLF4 all over the cytosol (Figure 5A). KLF4 is a transcription factor, and the nuclear localization of KLF4 indicates its active form. This further confirms that KLF4 is active in PED-tear-induced HCLE cells. With these gene expression data, we performed STRING analysis and showed the importance of these genes in regulating other crucial factors important in driving PED pathology (Figure 6). We also created a correlation plot to understand the positive and negative association of these factors in PED.

In summary, tears from PED patients can induce KLF4 production as well as TGFβ2 and MMP9 activities. This points out the dual nature of tear secretions in terms of containing factors that can affect corneal epithelial defect healing.

### 3.3. Epithelial Scrapes of Patients Indicate a Chronic Stage of Inflammation Associated with Compromised CE Homeostasis

Next, we checked for the overall gene expression changes in the corneal epithelial tissue in vivo. Tears contain only secreted factors, so other intra-cellular factors and cellular communications are not captured in tears. We collected epithelial scrapes from six PED patients and six healthy individuals going through PRK surgery. A list of important genes, including cellular communication genes (E-cadherin, desmoglein); genes related to the immune system (TNF α, IL6, IL8, and STING); and homeostasis markers (KLF4 and GPX4) were quantified using qPCR (Figure 6). Even though we observed significantly higher expression of TGFβ2 in PED-tear-induced HCLE cells, it was not detected in the corneal epithelial cells in vivo.

As expected, in healthy individuals as well as PED patients, both groups had intra-group differences in gene expression, but interestingly, the gene expression levels in the control group had lower deviations. However, E-cadherin, desmoglein, and GPX4 were significantly downregulated in the PED group. KLF4 is also downregulated in the PED group (*p* < 0.1), contrary to the increased expression of KLF4 in HCLE cells treated with PED tear. On the other hand, IL6 (*p* < 0.1), IL8, and TNFα (*p* < 0.05) were highly upregulated in the PED group. All the PED patients had defects for at least 4 weeks in this cohort while recruited in the study, and they should normally not be in the inflammatory stage. This strongly suggests that inflammatory cytokines- TNFα, IL6, and IL8 are likely to be responsible for the persistent defect in the cornea. Poor cell–cell adhesion is also observed in PED patients, as seen in the lower levels of desmoglein and E-cadherin.

### 3.4. Correlation of In Vivo Results with Clinical Factors

**Figure 7:**
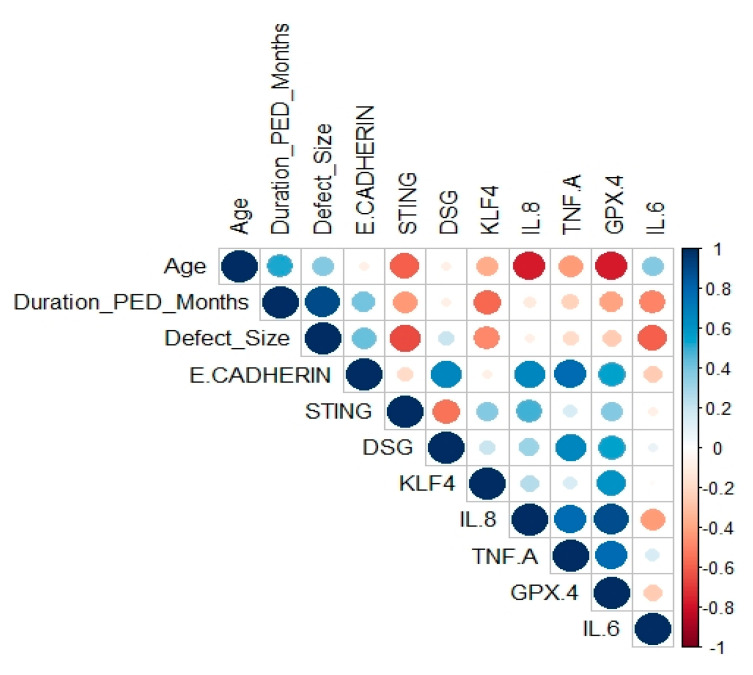
Correlation plot of qRT-PCR results and few clinical features. Intensity of blue color represents stronger positive correlation, whereas intensity of red color represents stronger negative correlations. The area of each bubble corresponds to the correlation coefficient.

Important clinical features (such as duration of PED, defect size, and age) were noted for all the patients. Thus, we were interested to find out if these features had any effect on gene expression in CE tissue. Pearson correlation was calculated for the quantitative clinical features (Figure 7) with the major relative in vivo mRNA expression level. Relative expression of STING, IL8, and GPX4 had strong negative correlations with age, i.e., patients of a higher age had lower expression of these genes in our patient group (Figure 7). The duration of PED while recruiting the patients and the defect size in the patients were highly correlated, as expected. Indirectly, this also corresponds to the severity of the PED. This severity had a strong negative correlation with the mRNA expression level of KLF4, STING, and IL6. E-cadherin and desmoglein were positively correlated, essentially indicating that the expression of both genes is affected similarly. Additionally, IL8, TNF-alpha, and GPX4 were positively correlated with each other.

## 4. Discussion

Management of patients with PED of the cornea can be challenge to even a seasoned ophthalmologist. Therefore, it is essential to understand not only the pathophysiology of poor epithelialization, migration, and appropriate closure of the wound, but also the mechanism of cellular and molecular changes taking place in the patient surface microenvironment. In this study, we demonstrated that the corneal epithelial cells have the ability to express proinflammatory factors in the presence of tears that could be a potential player in PED pathology [17]. A corneal surface needs a viable cell source of CE, as well as a conducive microenvironment, to generate healthy CE, thereby maintaining the delicate balance of proliferation, migration, and differentiation.

In this study, we focused on the role of tears and the PED scrapes as a base material to understand the disease pathology by profiling the gene expression. We used gene expression as the input data to establish correlation and the possible biological pathways involved in PED. Tears have been extensively studied in various ocular surface diseases as a direct or a surrogate indicator of the tissue state [12,18]. In several eye inflammatory disorders, tears have been analyzed and proposed as a source of pro-inflammatory factors, and their secretion is guided by the disease and the micro-environment milieu [12,19]. In our study, we found that using tears treatment in HCLE cells resulted in increased expression of pro-inflammatory markers such as IL6, IL8, TNF-alpha, and fibrotic marker TGF-ß2, indicating that tears are enriched in factors that have the potential to initiate an inflammatory response. In addition to evaluating the gene expression, we also observed increased expression of active MMP9 in the tears-treated HCLE cells supernatant. Studies report wound fluid levels of MMP9 at initial presentation in the clinic as an important predictor of the healing outcomes in diabetic foot ulcers [20]. We also observed increased expression of transcription factor KLF4 at the transcript level in cells treated with tears. Immunofluorescence revealed that the PED tears treatment induces translocation of KLF4 in the nucleus compared to the control, where KLF4 staining was seen in the cytoplasm as well as the nucleus, indicating that PED tears could potentially induce transcriptional activation of KLF4. To confirm the transcriptional activation, we evaluated the direct targets of KLF4; beta-catenin and Desmoglins expression increased, as expected, but E-cadherin expression decreased, which is contrary to what is known in the literature. Increased expression of KLF4 is associated with cell cycle arrest and apoptosis [21,22,23]. We also observed increased expression of GPX and SLC7A11 and a marginal decrease in NCO4 in the HCLE cells treated with tears, indicating that tears treatment induces oxidative stress, resulting in increased expression of these enzymes. Therefore, tears play an important role in PED by inducing the expression of pro-inflammatory cytokines. Increased expression of KLF4 coupled with translocation to the nucleus could be the initial step in the development of PED.

In order to understand that tears are a crucial player in PED, we evaluated the gene expression in the CE of PED patients scrapes which were continuously nourished by tears. We were interested in understanding the levels of the same cytokines as seen in the tears-treated HCLE cells. The profile of pro-inflammatory cytokines was in line with the tears-treated HCLE cells gene expression. Surprisingly, IL6 levels were very high in PED patients’ epithelium compared to that of the control. On other hand, the expression of KLF4 and its target genes expression decreased, contrary to what we saw in tears-treated HCLE cells. IL6 is known to have an important role in wound healing [24]. It acts as a neurotrophic factor [25] and its expression is increased in HSV1 infection [26,27]. As the patients recruited in this study showed HSV1-mediated PED, increased IL6 expression is an expected result, and it also points to the central role of IL6 in PED pathology. We also observed decreased expression of two enzymes that are protective against oxidative stress: GPX and SLC7A11. The decreased expression of KLF4 is associated with loss of CE epithelial phenotype, compromised barrier function, tight junctions, and adherent junctions essential for maintaining CE homeostasis [21,22,23,28].

### Discussion of Correlation Plot

Even with only six PED patients, we showed how the clinical features are correlated with the gene expression in vivo. As it is known that age is one crucial factor for immune regulations, it is seen in the PED patients that expression of immune genes (IL8, TNF-alpha, STING) was lowered in patients of a higher age. Interestingly, expression of IL6 was not found to be strongly related to age; rather, IL6 expression was negatively correlated with defect size. This indicates that IL6 is expressed more in the early phase of PED and when the defect size is smaller. Additionally, IL6 expression was not related to any other immune genes investigated in this study (STING, IL8, TNF-alpha). This opens up the possibility that IL6 may be a crucial gene in the development of PED in its early phase, and could thus be a potential target. However, this must be validated in a higher sample size and through an in-depth understanding of the disease mechanisms.

Another interesting gene is KLF4, which was found to be negatively correlated with the severity of PED. The higher the severity, the lower the expression of KLF4. As Tiwari et al. [21,22,23,28] have demonstrated, KLF4 is important in maintaining the homeostasis of corneal epithelial cells. This indicates that lower expression of KLF4 is another important key factor for PED development.

**Figure 8:**
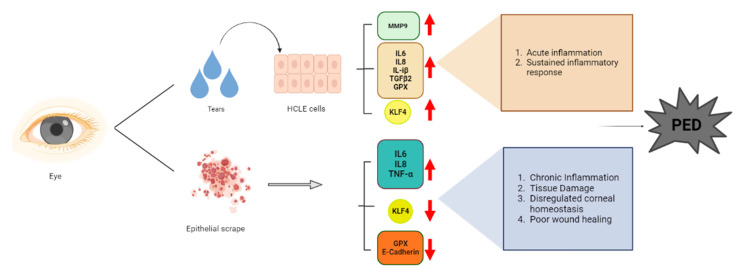
Schematic representing the summary of the project using BioRender.

In conclusion, tears play an important role in PED pathology, setting up a pro-inflammatory milieu which, coupled with increased KLF4 (an inhibitor of the cell cycle), could be the potential driver of PED disease pathogenesis. (Figure 8 shows a summary of this study). However, the epithelial scrape is an indicator of the chronic stage of PED. Knowing this, our study will provide insight into the cross-talk between HCLE and KLF4. Investigating the downstream targets in PED samples will provide an avenue for evaluating these as potential therapeutic targets.

## Figures and Tables

**Table 1:** Clinical and demography details of the patients included in the studies.

Patients	Age	Sex	Clinical Diagnosis	Penetrating Keratoplasty (PK) Status	Duration PED Months	Defect Size (mm^2^)
PED1	66	M	HSV	POST-PK	1	2
PED2	44	M	HSV	POST-PK	2	6
PED3	40	M	HSV	POST PK	1	12
PED4	70	M	HSV	NO	10	49
PED5	65	M	PSEUDOMONAS	POST PK	4	36
PED6	78	F	PSEUDOMONAS	NO	3	35

## Data Availability

Not applicable.
